# A Pathetic Scene

**Published:** 1890-07

**Authors:** 


### A PATHETIC SCENE.

"If there was any doubt in my mind of a hereafter, of a heaven, and of a Savior," said a member of the Cogburn Club, at Utica, N. Y. recently, "it would be utterly dispelled by an incident which occurred recently under my observation. The happy family of Edward W. Hill was entered by the angel of death, and both his little ones carried off. The first was Lillian, a sweet, plump, pretty baby. Within a month, her sister Harriet, a lovable, handsome little girl, barely turned four years of age, was stricken down. On her death bed, while the little limbs were growing colder and colder, and the dew was gathering on the white forehead, the little face took on a look of calmness, transfiguring it, the blue eyes opened with a smile, and the child, in a voice fairly ringing with joy, cried : 'Oh ! mamma, look at them coming ! See the angels coming ! And look, there's Lillian in the front. Mamma, I am going to meet her,' and the soul went out into a brighter, better country with Lillian, and the shining host of heaven. It was a balm to that mother's bleeding heart, and the assurance, the knowledge that she has treasures in heaven, did much to reconcile her to the severe loss. Don't you believe that the gates of the golden city were opened to that little one's eyes as a comfort to that mother? Can any one with such a proof doubt the existence of a home beyond the grave? I tell you it dashes to pieces all the castles of oratory builded by the Paines of the past, and refutes the eloquent sophistries of the Ingersolls of the present."

